# Determination of Behavioral Changes Associated with Bovine Respiratory Disease in Australian Feedlots

**DOI:** 10.3390/ani13233692

**Published:** 2023-11-29

**Authors:** Brad J. White, Dan R. Goehl, Joe P. McMeniman, Tony Batterham, Calvin W. Booker, Christopher McMullen

**Affiliations:** 1 Precision Animal Solutions, LLC, Manhattan, KS 66502, USA; 2 Meat and Livestock Australia Limited, North Sydney, NSW 2059, Australia; 3 Apiam Animal Health, Bendigo, VIC 3554, Australia; tony.batterham@sydney.edu.au; 4 TELUS Agriculture, Box 140, Okotoks, AB T1S 2A2, Canada

**Keywords:** beef cattle, bovine respiratory disease, remote early disease identification, shipping fever

## Abstract

**Simple Summary:**

Accurately identifying bovine respiratory disease is challenging in feedlots, and several studies have highlighted the importance of monitoring behavior to determine cattle health status. The objective of this study was to describe individual differences in physical activity, feeding/watering patterns, and social behavior when associated with health status in commercially raised beef cattle during the first 28 days on feed. Data from a previous study monitoring cattle behavior and associated health outcomes at two Australian feedlots were used to determine potential associations of interest. These data illustrated an association between several key behavioral parameters (physical activity, proximity to feed and water, and social behavior) and health status categories (healthy vs. sick), and many of these effects were modified by the time of day or days spent at the feedlot. Sick cattle were less active, spent more time by themselves, had differing attendance at the feed bunk across days on feed, and spent more time near water and the feed bunk at certain times of the day. Sick cattle were also more social early in the feeding period but less social as time progressed. These behavioral observations may provide information to help inform the process of diagnosing cattle with bovine respiratory disease.

**Abstract:**

Accurately identifying bovine respiratory disease is challenging in feedlots, and previous studies suggest behavioral monitoring is important. The study objective was to describe individual differences in physical activity (distance traveled), feeding/watering patterns (proximity to feed and water), and social behavior (average cattle within 3 m) when associated with health status in commercially raised beef cattle during the first 28 days on feed. Data from a previous Australian feedlot study monitoring cattle behavior and associated health outcomes were analyzed. Health status categories were generated for all cattle, and each animal was categorized as known healthy (HLTH), known diseased (SICK), or intermediate/uncertain (INTR). The INTR animals were excluded from the final analysis. Key findings included: differentiation in activity between SICK (*n* = 138) and HLTH (*n* = 1508) cattle dependent on time of day, SICK cattle spending more time in water and feeding zones early in the feeding phase (<6 days on feed), SICK cattle spending more time in the water and feeding zone during the overnight hours, and SICK cattle spending more time in groups early in the feeding phase but more time in isolation after the first week on feed. Results illustrate behavioral data were associated with important health outcomes.

## 1. Introduction

Diagnosis of bovine respiratory disease (BRD) is a major challenge in the beef industry, and BRD identification is commonly based on subjective assessments with relatively poor accuracy [1,2]. Misclassification of diseased cattle is relatively common using current BRD diagnosis techniques [3]. The diagnostic sensitivity and specificity of identifying diseased cattle based on signs of clinical illness for BRD have previously been reported to be as low as 61.8% and 62.8%, respectively [2]. In simple terms, ~38% of diseased calves in a population may go undiagnosed, and ~37% of calves diagnosed with BRD may be misdiagnosed when they are actually not diseased [2]. Multiple studies have evaluated methods to diagnose BRD more accurately in commercial settings, including blood parameters, temperature monitoring devices, and thoracic ultrasound [3,4,5,6,7].

Many of these diagnostic systems are designed primarily to confirm diagnosis after an animal is preliminarily identified with BRD (e.g., thoracic auscultation, thoracic ultrasound, etc.). These systems could help improve the specificity of the BRD diagnosis in the population preliminarily identified as potentially ill. Other diagnostic systems focus on remotely monitoring cattle in real time to identify sick cattle in a normal housing environment (e.g., ruminoreticulum boluses, behavioral monitoring, etc.), and these systems may have the potential to identify new cases not observed as ill. In a review of several available monitoring systems, the authors noted that one of the more promising directions for improving BRD diagnosis is the utilization of remote monitoring of behavioral changes [3]. Whereas the use of remote monitoring technologies in beef cattle is still a relatively new field of interest, there has been a significant amount of research done in dairy cattle. Although the day-to-day routines and life of dairy and beef cattle differ greatly, there are common behavioral measures that may be useful in the diagnosis of disease in both types of animals. Physical activity (distance traveled or steps taken/lying time), feeding and watering behaviors, and social behaviors have all been suggested as potential indicators of cattle health status in both dairy and beef cattle [8,9,10,11,12,13,14,15,16,17,18]. Furthermore, these behavioral variables have been directly linked to predicting BRD [8,9,11,19,20,21]. Previous evaluations of behavioral monitoring have illustrated the ability to find BRD cases prior to visual observation with greater accuracy, which may influence treatment success [22,23,24,25].

Behavior varies among individual animals, and understanding the drivers of differences between cattle may help delineate signs of illness. A more refined understanding of behavioral changes may also allow remote monitoring systems to be more focused on the accurate detection of BRD. Therefore, the objective of this study was to describe individual differences in physical activity (distance traveled), feeding/watering patterns (proximity to feed and water), and social behavior (average cattle within 3 m) when associated with health status in commercially raised beef cattle during the first 28 days on feed (DOF). All of these behaviors were expected to change based on an animal’s health status.

## 2. Materials and Methods

### 2.1. Trial Overview

The current trial used observational data collected from a clinical trial conducted at two feedlot locations in Australia designed to compare potential differences between BRD identification methods (the Remote Early Disease Identification [REDI] system and conventional visual observation; manuscript under preparation). The REDI system is a system used to continuously monitor behavior and potentially identify disease [23]. This system utilizes cattle behavior data to inform a proprietary algorithm that identifies cattle that are suspected to have BRD [23]. The clinical trial consisted of feeding beef steers in 12 pens for each method of BRD identification (six pens per method at each location). Each study site procured its own cattle, and typical Australian buying practices were used to procure cattle from sale yards for site 1 and direct vendor procurement for site 2. Breeds at site 1 were Angus, Angus cross, *Bos indicus* cross, British Cross, European, Hereford, Murray Grey, and Shorthorn. Breeds at site 2 were Angus cross, Hereford cross, and Shorthorn Cross. A total of 3114 cattle were observed using the REDI system (site 1 *n* = 1800 animals, site 2 *n* = 1314 animals). There were six pens at each site, with 300 animals in each pen at site 1 and 216 to 223 animals in each pen at site 2. Each pen had 50 m of bunk length and 58 m of depth (22.7 cm per animal of bunk space, or 12.3 m^2^/animal), with a 3% slope. All pens had a single strip of shade cloth (11 m wide) in the middle of the pen. Pen waterers were shared along the fence line with adjoining pens and cleaned weekly. Cattle were fed up to two times per day, with the first feeding occurring between approximately 7 and 11 am.

Cattle in the REDI pens that were identified by the system as positive for BRD using the same criteria as previous work were treated [23]. These criteria include monitoring behavioral changes from baselines for individual animals. After a targeted 60 DOF, all cattle were monitored using visual observation for the remainder of the feeding period. Trained operational personnel at each location evaluated cattle daily for visual signs of BRD, including increased respiration, anorexia, and depression. Following visual identification in the pen, cattle were transported to a hydraulic chute for further examination and were treated if BRD was confirmed. Cattle were slaughtered at approximately 114 DOF for site 1 and 187 DOF for site 2.

### 2.2. Individual Animal Behavioral Data

Cattle were monitored with REDI to determine behavioral patterns. The system consisted of a REDI tag placed in the ear of each animal that communicated with readers surrounding the perimeter of the pen. The hardware was a commercially produced system (Smartbow, Vienna, Austria). Communication between tags and readers allowed the location engine software to calculate the location of each animal within the pen (accurate to within 0.5 m) at approximately 4 s intervals. Location data were transferred to the readers that moved data to an on-site server, which calculated activity, proximity, and social indices using previously described methods [22,23]. The cloud server then utilized the REDI disease classification engine [22,23] to generate reports containing the BRD status of each individual steer. The reports were then relayed to a digital platform for personnel to determine which steers needed to be pulled by pen riders for daily treatments.

The current trial focused on the raw data collected by the REDI system to describe cattle behavior during the first 28 DOF. Key behavioral components were selected to describe cattle behavior in three areas: activity (distance traveled), proximity to areas of interest (percent time in feeding and water zones), and social index (average cattle within 3 m). These three areas of behavior provide an overview of individual animal movement within the pen, time spent near eating and drinking facilities, and time spent around other cattle within the pen. The feeding and water zones were identified when an animal was within 1 m of the designated feeding or watering area. These values indicate proximity but not necessarily that the animal was actually eating or drinking. The decision to analyze data from the first 28 DOF was based on the experience of the authors as it pertains to cattle behavior at the feedlot. There is little to no data in the literature that definitively describes the time period during which cattle behavior is most dynamic at the feedlot, and there is no scientific consensus on the time period during which cattle are most at risk for developing BRD. However, the authors have anecdotally observed through previous research and unpublished foundational work developing the REDI system that cattle behavior appears to be the most dynamic during the first 28 DOF due to a wide variety of factors that have yet to be fully elucidated. Therefore, only behavioral data collected during this time period was analyzed for the purposes of this manuscript.

Data cleaning consisted of removing unrealistic values that may have occurred due to tag losses or hardware issues. Only values considered unrealistic were removed from each behavioral outcome. Unrealistic values may have resulted from inaccurate data reported from the tag, and cutoffs for levels of unrealistic data were determined by the authors. For distance traveled, values ranged from 0 to 750 m, with 12,946 hourly observations (0.35%) that were greater than 750 m out of 3,651,122 hourly observations removed. The percentage time in the feeding and water zones ranged from 0 to 100%, and 28 hourly observations (<0.01%) for each variable outside of these ranges were removed. The average number of cattle within 3 m ranged from 0 to 15, with 180 hourly observations (<0.01%) outside this range removed.

### 2.3. Pulmonary Data at Harvest

Data were limited to include only cattle that died of BRD or received a lung or pleurisy score at harvest. Overall lung scores consisted of monitoring pulmonary consolidation and pleurisy scores. Scoring was performed by the same three people who were trained prior to initial scoring. Pulmonary consolidation was recorded based on a scale of 0 to 100%, similar to previous work [26]. Consolidation scores were categorized into three categories: 0 to 1%, 2 to 9%, and 10 to 55%. Pleurisy scores ranged from 0 to 3. A score of 0 was no pleurisy, a score of 1 was pleuritic fibrinous attachments between lung lobes or on the lung surface with no adhesion on the pleura of the thorax, a score of 2 was pleuritic lesions with localized adhesion to the thoracic wall, and a score of 3 was severe pleuritic adhesions with the chest requiring “stripping” or additional removal, as adhesions between the lungs and plural cavity were extreme. In cases where the lungs were adhered to the thoracic wall, a pleurisy score of 3 was given, but a consolidation score was not recorded (reported as NS). Animals that died or were rejected from the trial did not have a lung score recorded. Animals that were condemned also did not have a lung score recorded. If an animal’s lungs were stuck to the thoracic wall, a consolidation score was not recorded, but a pleurisy score was reported. An overall pulmonary disease score was created based on the combination of consolidation and pleurisy scores.

### 2.4. Production Data

Not all animals in the dataset had an individual live weight prior to slaughter; however, a hot carcass weight was available. The average dressing percentage on available data of live weight and hot carcass weight was calculated to be 58% (based on AUS-MEAT hot standard carcass weight trim). This percentage was used to calculate an estimated final live weight for all cattle that were shipped for slaughter (even if live weight was available, to maintain consistency). The estimated final live weight, initial body weight, and DOF were used to calculate an average daily gain (ADG), or how much weight cattle gained per day, over the entire period. Animals that died were not included in ADG calculations.

### 2.5. Health Status Categorization

Health status categories were generated for all cattle, and each animal was placed into one of three categories: known healthy (HLTH), known diseased (SICK), or intermediate/uncertain (INTR). Cattle were classified based on their known individual overall health outcomes to provide a case definition for comparison of behavioral characteristics. Cattle were placed in the HLTH category if they had no lung or pleurisy score greater than 1, had never been treated for BRD, did not die, and were not rejected for any reason during the trial. Cattle were placed in the SICK category if they were classified as having a lung or pleurisy score of 3 (which was collected at slaughter), died due to BRD, or were rejected from the trial due to BRD. All other cattle were placed in the INTR category. Identification of BRD by the REDI system was not an inclusion criterion for any of the three health status categories. Although REDI was used to identify cattle for initial BRD treatment, follow-up evaluation by trained animal health personnel was required to confirm a BRD relapse. The decision to treat an animal multiple times was ultimately based on clinical diagnosis. This also applied to the decision to reject an animal for BRD, which was made based on an evaluation by trained animal health personnel. Animals could also never be treated for BRD but still qualify for the SICK category based on post-mortem examination and having a lung or pleurisy score of 3.

### 2.6. Statistical Analysis

Data handling and statistical analysis were performed using R in RStudio. Descriptive analyses were performed on the final individual animal behavioral dataset. Individual animal behavioral data was aggregated to individual animals over the period and by hour throughout each day. For the hourly analysis, data were evaluated using all the hours for each animal during the period, comparing variability over time. For the individual animal analysis, data were aggregated to the animal level over the entire trial period. A data subset was created containing only cattle in the HLTH and SICK categories, as these case definitions clearly delineated health status. In this subset, initial body weight was limited to 300 to 500 kg and categorized by 50 kg groups to avoid linearity assumptions with behavioral outcomes. Animals in the INTR category were not included, as many of them had conflicting indications of disease (e.g., pulmonary lesions present but never identified with disease or no pulmonary lesions but identified and treated for disease). Analyzing only the HLTH and SICK categories allowed for inferences of behavior with known health outcomes. Multivariable statistical models (RStudio:lmer) were created to evaluate relationships between key behavioral variables and potential covariates of interest, including the fixed effects of health status (HLTH/SICK), DOF, hour of the day, initial body weight category, and potential interactions between health status and DOF, and health status and hour of the day. Each statistical model accounted for a lack of independence among observations with random effects due to study site, cohort, and repeated measures on individual animals. Independent statistical models were created for the first 28 DOF to determine potential associations between covariates and each behavioral variable. Only variables with a significant (*p* < 0.05) association with the key behavioral outcomes of interest were reported. Comparisons were made among health status groups within DOF or record hours using the Tukey adjustment for multiple comparisons (*p* < 0.05).

## 3. Results

### 3.1. Health Status Categorization

Data were limited to the 3,114 animals monitored with the REDI system for behavioral analysis and comparison. These behavioral data contained 3,651,122 observations (hourly summaries). There was an average of 48.9 days of behavioral monitoring per animal. A combination of lung scores, treatment history, and health outcomes were used to categorize animals based on health status. Animals in the SICK category (*n* = 138 animals) were rejected due to BRD (*n* = 10 animals), died due to BRD (*n* = 8 animals), were treated twice for and subsequently rejected due to BRD (*n* = 14 animals), had a lung score of 3 (*n* = 3 animals), or had a pleurisy score of 3 (*n* = 103 animals). Animals in the HLTH category (*n* = 1508 animals) received no treatment for BRD and did not have a pleurisy or lung score greater than 1. Animals in the INTR category (*n* = 1468 animals) encompassed the remainder of the dataset. The distribution of health status categories by study site and pen is displayed in Table 1.

Animals designated to the SICK category had lower weight at REDI tag removal, final estimated live weight, and hot carcass weight compared to animals in the HLTH category (Table 2). These lower weights translated to decreased ADG from arrival to both the time of REDI tag removal and shipment for slaughter in the SICK animals compared to the HLTH animals.

### 3.2. Distance Traveled and Health Status

**Figure 1:**
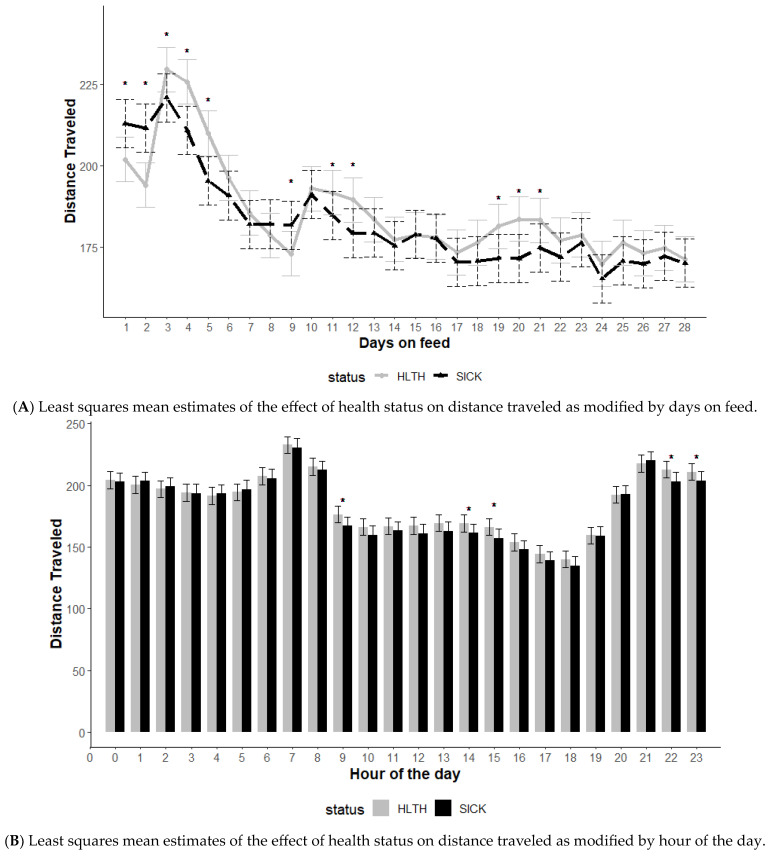
Least squares mean distance traveled from the generalized model with fixed effects for the interaction between health status and days on feed (**A**) and health status and hour of the day (**B**). The model included effects to control for clustering due to repeated measures on individual animals within the pen and study site. Each * signifies a significant difference (*p* < 0.05) within the day or record hour between health status categories.

The effect of health status on distance traveled was modified by DOF and hour of the day (*p* < 0.01) but not the initial body weight category (*p* = 0.56) (Figure 1). Data included all SICK and HLTH animals as identified in each health status category regardless of when or if the animal had been identified as diseased. The SICK animals displayed greater distance traveled relative to HTLH animals over the first 2 DOF, then lower distances traveled on days 3, 4, and 5 (Figure 1A). An interaction between record hour and health status category was observed (*p* < 0.01), with healthy animals traveling higher distances at hours 9, 14, 15, 22, and 23 (Figure 1B).

### 3.3. Time near Water and Feed Associated with Health Status

**Figure 2:**
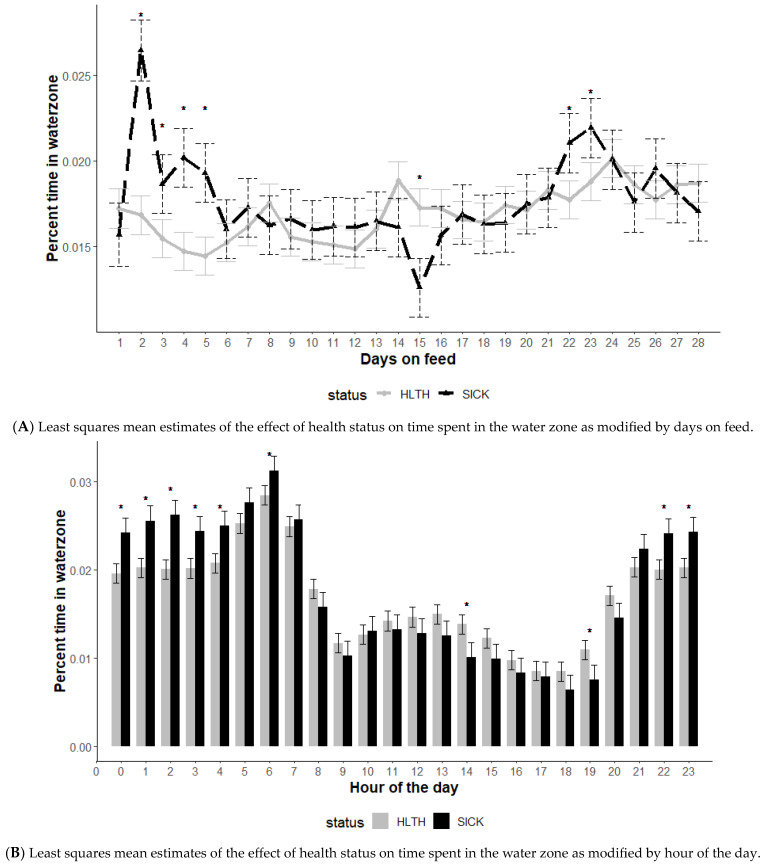
Least squares mean time spent in the water zone from the generalized model with fixed effects for the interaction between health status and DOF (**A**) and health status and hour of the day (**B**). The model included effects to control for clustering due to repeated measures on individual animals within the pen and study site. Each * signifies a significant (*p* < 0.05) difference within the day or record hour between health status categories.

The effect of health status on time spent in the water zone was modified by DOF and hour of the day (*p* < 0.01) but not the initial body weight category (*p* = 0.15) (Figure 2). The average percent of time spent in the water zone illustrated several differences among animals classified as HLTH compared to those classified as SICK. Early in the feeding phase (2 to 5 DOF), animals in the SICK category spent a greater percentage of time at the water compared to those in the HLTH category (Figure 2A).

The average time spent in the water zone showed differences in watering behavior between the SICK and HLTH categories based on time of day (Figure 2B). Animals in the HLTH category spent more time at the water compared to SICK animals during mid-morning to late afternoon (10 am to 8 pm). However, during night-time hours, SICK animals spent more time at the water.

**Figure 3:**
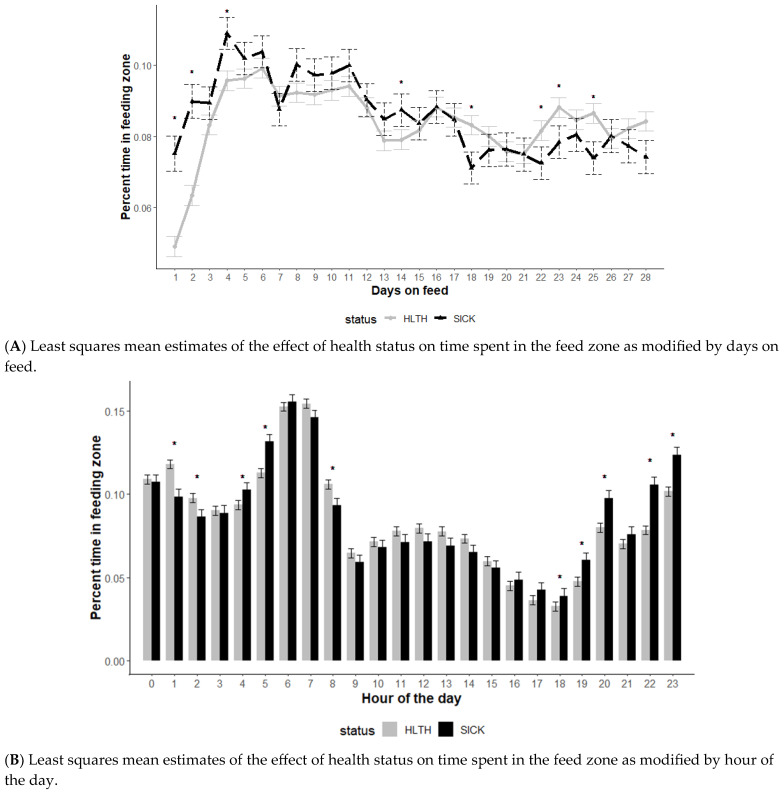
Least squares mean time spent in the feed zone from the generalized model with fixed effects for the interaction between health status and DOF (**A**) and health status and hour of the day (**B**). The model included effects to control for clustering due to repeated measures on individual animals within the pen and study site. Each * signifies a significant (*p* < 0.05) difference within the day or record hour between health status categories.

The effect of health status on time spent in the feed zone was also modified by DOF and hour of the day (*p* < 0.01) but not the initial body weight category (*p* = 0.61) (Figure 3). Similar to water behavior, animals designated as SICK spent more time in attendance at the feed bunk early in the feeding period (DOF 1, 2, and 4) and less time later in the feeding period (DOF 18, 22, 23, 25) (Figure 3A).

Feeding behavior differed by SICK and HLTH animals based on the hour of the day monitored (Figure 3B). During the lower activity evening and early morning hours (hours 18, 19, 20, 22, 23, 04, 05), SICK animals spent more time in the feeding zone (*p* < 0.05), with fewer differences noted during the primarily daytime hours (7 am to 5 pm).

### 3.4. Social Indices and Health Status

**Figure 4:**
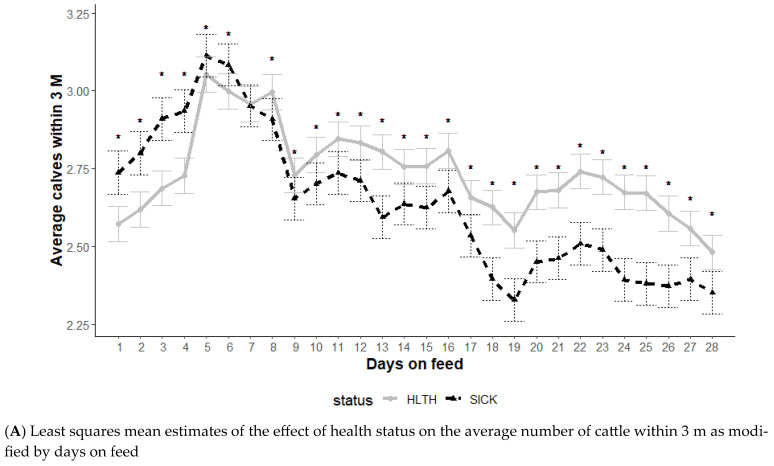

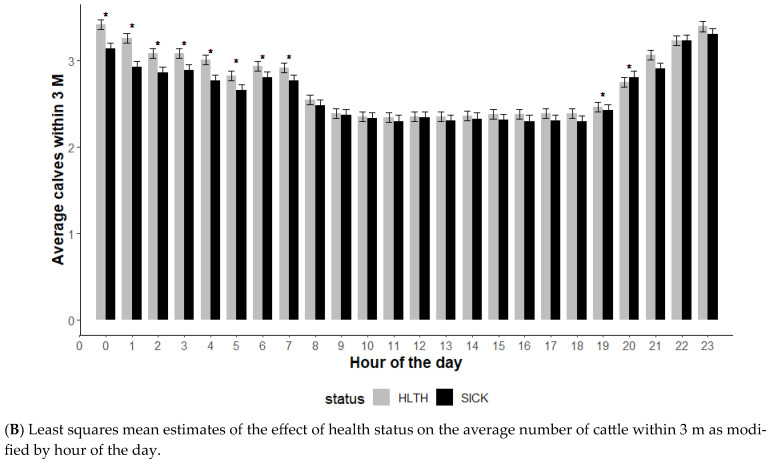
Least squares mean average number of cattle within 3 m from the generalized model with fixed effects for the interaction between health status and DOF (**A**) and health status and hour of the day (**B**). The model included effects to control for clustering due to repeated measures on individual animals within the pen and study site. Each * signifies a significant (*p* < 0.05) difference within the day or record hour between health status categories.

The average number of animals within 3 m is an indication of socialization, and the effect of health status on animals within 3 m was modified by DOF and hour of the day (*p* < 0.01) but not the initial body weight category (*p* = 0.12) (Figure 4). The average number of cattle within 3 m illustrated a distinct relationship with health status (Figure 4A). Until 6 DOF, the SICK animals had higher numbers of other cattle within 3 m (more social), but this changed from day 8 through day 28 when the HLTH animals had a higher number of cattle within 3 m (Figure 4A).

Time of day had some effect on the differences in social behavior based on health status, with no differences noted during mid-day (*p* ≥ 0.05) and some differences noted in the overnight hours (*p* < 0.05). Animals in the SICK group spent more time with less cattle within 3 m during the overnight hours (midnight to 7 am).

## 4. Discussion

The objective of this study was to describe individual differences in physical activity, feeding/watering patterns, and social behavior when associated with health status in commercially raised beef cattle during the first 28 DOF. Health status was shown to have a significant impact on distance traveled (physical activity), time near water and feed, and social behavior. Moreover, many of these effects were modified by time of day and DOF, highlighted by novel observations in cattle deemed to be clinically sick with BRD.

Physical activity and feedlot performance are associated with cattle health status categories in the current study. Unsurprisingly, animals designated to the SICK category had poor performance compared to those in the HLTH category. These findings align with previous research [27,28,29] and indicate that the HLTH and SICK categories are correctly delineating degrees of illness. However, these findings do not tell the entire story, as the activity of SICK animals differed by time of day and DOF. It is unclear why SICK animals traveled a greater distance than HTLH animals over the first 2 DOF and whether this observation is truly a function of the health status category, but previous work has illustrated lower activity in calves with BRD. Many of the behavioral changes identified in other research occurred prior to the time of visual diagnosis [8,19].

Similar to DOF, the time of day also appeared to modify cattle behavior. One observation to note is cattle appear to have high-activity time periods (between approximately 7 to 9 am and 9 pm to midnight). During these time periods, few differences were identified in the morning hours typically associated with feeding time. This may be due to social pressures being highest during these time periods when even SICK cattle will move with the group. During low-activity times (mid-day), some separation (hours 9, 13, 14) in activity levels between the HLTH and SICK animals was observed. One conclusion may be that the best time to try to observe potential differences in health status is likely a time when baseline activity levels are lower and social pressures are not elevated. Interestingly, the HLTH animals appeared to be more active closer to midnight than the SICK animals. It is unclear what drove this difference, although reasonable speculation might suggest sick animals tend to rest more during less social time periods of the day.

Time near water may provide valuable behavioral information for diagnosing BRD, but time of day and DOF must be considered when interpreting these observations. It is noteworthy that SICK animals spent a greater percentage of time at water compared to those in the HLTH category. This could be due to several potential factors, including acclimatization to the new environment and the cattle entering the facility at some level of dehydration, which may have increased water consumption. Previous research illustrates that shrink, or weight loss during transit (often associated with changes in hydration status), is associated with increased BRD risk [30]. One caveat to consider is the REDI system only measured water attendance, not consumption; however, previous research has illustrated that, when monitored by a real-time location system compared to video observation, the probability of cattle drinking when in attendance is 42–54% [31]. There was also a distinct difference between SICK and HLTH animals regarding the hours of the day during which they spent time in the water zone, with HLTH animals spending more time near water during the day-time hours and SICK animals spending more time during night-time hours. One theory based on past observations is that watering behavior during the day may be a more social event, whereas in evening hours it is based on individual animal preferences. This finding indicates a simple threshold based on time near the location of water may not elucidate the differences in health status based on these behavioral patterns.

Time near feed may also provide valuable behavioral information for diagnosing BRD depending on the time of day and days after arrival. Similar to water behavior, though, time of day and DOF must also be considered when interpreting these observations. Cattle designated as SICK spent more time in attendance at the feed bunk early in the feeding period. The feeding area may be an area for socialization and maintaining herd cohesion, which may be driving the difference in behavior. However, cattle may be trying to participate in herd activities and hide potential illness early in the disease process, leading them to spend more time at the feed bunk. Again, the time of day also appeared to modify feeding behavior. Observation of anorexia or non-aggressive feeding behavior is often considered a hallmark for visual BRD diagnosis and has been investigated in dairy cattle as a potential variable of interest in predicting health status [18,21,32]. However, these results indicate that the timing of observations to identify potentially ill animals may be very important. During the highest feeding time activity hours (06, 07), no differences were noted based on health status, and few differences were identified during the day.

Social parameters provided an interesting insight into cattle behavior and how they might be used for diagnosing BRD, with differentiation between SICK and HLTH animals observed early in the feeding phase. Individual animal isolation or separation from the group is often considered a sign of BRD for visual observation [16], but in this study, SICK animals spent more time with other HLTH animals early in the feeding phase. One behavioral hypothesis is that, due to predator and prey relationships, diseased cattle are reluctant to separate from the herd, making them harder to identify [33]. These data may indicate that SICK cattle were ill early in the feeding phase and did not separate from the herd until after the disease had significantly progressed. Among the behavioral attributes monitored, socialization illustrated several significant differences based on health status. Time of day also appeared to have an effect on the differences in social behavior based on health status. Cattle in the SICK group spent less time with fewer cattle during the night-time and early morning, which would have been challenging to observe during this period without remote monitoring. This is especially noteworthy as isolation is commonly considered a hallmark of BRD.

Limitations of this work include the observational nature of the study, with behavioral changes evaluated relative to DOF after arrival. The included study data focused only on known outcomes (HLTH, SICK), which allowed for comparison between these two groups but does not account for cattle in the INTR group. Thus, the results should not be used as definitive predictors of or diagnostic criteria for BRD since there undoubtedly were cattle in the INTR group that were sick with BRD. The analysis did not evaluate time relative to BRD identification, meaning inferences could only be made about the HLTH and SICK cattle based on DOF. Since it is not known when cattle actually developed BRD, it is also unclear to what extent cattle behavior was influenced by the disease process and how it progressed. The external validity of the results is limited to the first 28 DOF at the feedlot. While not a major limitation, it is important to recognize that the data reported here cannot be generalized to cattle behavior at later stages of the feeding cycle. Due to the retrospective nature of the data, the authors were unable to draw any conclusions regarding the causation of observations and could only comment on identified correlations, which require further experimental investigation.

## 5. Conclusions

Several key behavioral parameters (physical activity, proximity to feed and water, and social behavior) were associated with health status categories in commercially raised beef cattle during the first 28 DOF at the feedlot. Time of the day and DOF modified some of these effects to varying degrees. These findings confirm behavioral dogma in identifying BRD in feedlot cattle, including observations that sick cattle are less physically active and spend an increased amount of time in solitude. However, there were additional findings that may help to provide greater context on animal behavior as it pertains to health status. The physical activity of sick cattle differed by time of day. There were unique times of the day during which sick cattle spent more time at the feed bunk. Watering behavior also differed early in the feeding phase for sick cattle. A differentiation in social behavior was observed early in the feeding phase, with sick cattle behaving more socially (more cattle within 3 m) until 6 DOF, after which they became less social. The behavioral observations reported here may provide additional information that can be used to help inform the process of diagnosing cattle with BRD, as well as improve the accuracy of precision monitoring systems in identifying BRD in feedlot cattle.

## Figures and Tables

**Table 1:** Distribution of cattle in each health category by study site and pen.

Site	Pen	HLTH Animals (% of Pen)	INTR Animals (% of Pen)	SICK Animals (% of Pen)	Total Animals
A	1	117 (39%)	171 (57%)	12 (4%)	300
A	2	112 (37.33%)	177 (59%)	11 (3.67%)	300
A	3	193 (64.33%)	102 (34%)	5 (1.67%)	300
A	4	230 (76.67%)	69 (23%)	1 (0.33%)	300
A	5	217 (72.33%)	76 (25.33%)	7 (2.33%)	300
A	6	166 (55.33%)	127 (42.33%)	7 (2.33%)	300
B	7	86 (39.09%)	116 (52.73%)	18 (8.18%)	220
B	8	88 (39.46%)	119 (53.36%)	16 (7.17%)	223
B	9	85 (39.53%)	117 (54.42%)	13 (6.05%)	215
B	10	99 (45.83%)	106 (49.07%)	11 (5.09%)	216
B	11	54 (24.55%)	150 (68.18%)	16 (7.27%)	220
B	12	61 (27.73%)	138 (62.73%)	21 (9.55%)	220
	Total	1508 (48.43%)	1468 (47.14%)	138 (4.43%)	3114

**Table 2:** Comparison of common performance characteristics among cattle in each health category using multivariable models. Superscripts within a row indicate statistical differences at *p* < 0.05. Each model contained relevant covariates of interest and effects to account for the lack of independence among data points. ADG = average daily gain, REDI = remote early disease identification.

Production Variable	HLTH		INTR		SICK		SE	*p*-Value
Initial body weight (kg/animal)	400.85	^a^	402.37	^a^	401.00	^a^	±34.04	0.49
Weight at REDI tag removal (~d 55; kg/animal)	515.26	^b^	513.89	^ab^	506.23	^a^	±5.42	0.03
ADG at REDI tag removal (~d 55; kg/animal/d)	2.21	^b^	2.18	^ab^	2.04	^a^	±0.2	0.04
Final estimated live weight (kg/animal)	663.22	^b^	661.34	^b^	650.67	^a^	±10.96	0.01
Final estimated live ADG (kg/animal/d)	1.74	^b^	1.74	^b^	1.54	^a^	±0.31	<0.01
Hot carcass weight (kg/animal)	384.66	^b^	383.57	^b^	377.39	^a^	±6.36	0.01
Meat Standards Australia Marbling score	377.93	^a^	380.25	^a^	380.47	^a^	±13.95	0.77
Ribeye muscle area	84.45	^a^	84.27	^a^	84.99	^a^	±1.32	0.58

## Data Availability

Data are contained within the article.
